# Horizontal Gene Transfer and Its Association with Antibiotic Resistance in the Genus *Aeromonas* spp.

**DOI:** 10.3390/microorganisms7090363

**Published:** 2019-09-18

**Authors:** J. Manuel Bello-López, Omar A. Cabrero-Martínez, Gabriela Ibáñez-Cervantes, Cecilia Hernández-Cortez, Leda I. Pelcastre-Rodríguez, Luis U. Gonzalez-Avila, Graciela Castro-Escarpulli

**Affiliations:** 1 Laboratorio de Investigación Clínica y Ambiental, Departamento de Microbiología, Escuela Nacional de Ciencias Biológicas, Instituto Politécnico Nacional, Carpio y Plan de Ayala, Col. Casco de Santo Tomás, Mexico 11340, Mexico; 2 Unidad de Investigación, Hospital Juárez de México, Av. Instituto Politécnico Nacional 5160, Magdalena de las Salinas, Gustavo A. Madero, Mexico 07360, Mexico; 3 Sección de Estudios de Posgrado e Investigación, Escuela Superior de Medicina, Instituto Politécnico Nacional, Salvador Díaz Mirón, Col. Casco de Santo Tomas, Mexico 11340, Mexico; 4 Laboratorio de Bioquímica Microbiana, Departamento de Microbiología, Escuela Nacional de Ciencias Biológicas, Instituto Politécnico Nacional, Carpio y Plan de Ayala, Col. Casco de Santo Tomás, Mexico 11340, Mexico

**Keywords:** antimicrobial resistance, horizontal gene transfer, *Aeromonas*

## Abstract

The evolution of multidrug resistant bacteria to the most diverse antimicrobials known so far pose a serious problem to global public health. Currently, microorganisms that develop resistant phenotypes to multiple drugs are associated with high morbidity and mortality. This resistance is encoded by a group of genes termed ‘bacterial resistome’, divided in intrinsic and extrinsic resistome. The first one refers to the resistance displayed on an organism without previous exposure to an antibiotic not involving horizontal genetic transfer, and it can be acquired via mutations. The latter, on the contrary, is acquired exclusively via horizontal genetic transfer involving mobile genetic elements that constitute the ‘bacterial mobilome’. This transfer is mediated by three different mechanisms: transduction, transformation, and conjugation. Recently, a problem of public health due to implications in the emergence of multi-drug resistance in *Aeromonas* spp. strains in water environments has been described. This is derived from the genetic material transfer via conjugation events. This is important, since bacteria that have acquired antibiotic resistance in natural environments can cause infections derived from their ingestion or direct contact with open wounds or mucosal tissue, which in turn, by their resistant nature, makes their eradication complex. Implications of the emergence of resistance in *Aeromonas* spp. by horizontal gene transfer on public health are discussed.

## 1. Introduction

Bacteria from the genus *Aeromonas* are microorganisms considered ubiquitous and native to aquatic environments, which can cause infections in humans and animals. It has also been shown that they can develop and spread antibiotic resistance in clinical and environmental settings [1]. Recently, several new species of *Aeromonas* have been identified, with *A. hydrophila*, *A. veronii*, *A. caviae*, *A. sobria*, and *A. salmonicida* being the most representative for their pathogenicity in humans and aquatic organisms [2,3,4,5]. The *Aeromonas* genus compromises 36 species [6] of short rod-shaped, non-spore-forming, Gram negative bacteria of approximately 1–3 µm in length [7]. The genus is part of the Gammaproteobacteria class in the order of Aeromonadales and it belongs to the Aeromonadaceae family [8]. These bacteria are catalase and oxidase positive, capable of fermenting glucose and liquate gelatin, and uncapable of fermenting inositol. They are described as facultative anaerobes and can tolerate elevated concentrations of sodium chloride (0.3–5%) [9]. They can produce a diverse variety of extracellular hydrolytic enzymes, such as arylamidases, esterases, amylases, elastases, deoxyribonucleases, chitinases, peptidases, and lipases [10]. They display an optimal growth temperature in the range of 22–35 °C, and can resist pH ranges from 4.5 to 9 [11].

The increase on the concentration of antibiotics in the environment creates a selective pressure to resistance genes, particularly in human waste streams in which resistance genes are released and mixed with antibiotics and other biocidal agents [12]. The exposure of environmental microorganisms to this mixture stimulates the fix of horizontal gene transfer (HGT) events, disseminating genetic resistance elements in several strains and species, increasing the microorganism’s abundance and penetration in new niches and hosts [13].

Considering that infections caused by bacteria of the genus *Aeromonas* are often difficult to eradicate due to their intrinsic resistance to β-lactam antibiotics such as ampicillin (especially, *A. media* and *A. caviae* are sensitive to this antibiotic). This resistance is due to the constitutive expression of AmpC β-lactamases combined with a low permeability of the external membrane and their noteworthy capacity to acquire resistance genetic elements to multiple groups of antimicrobial agents.

Several research groups have demonstrated that environmental bacteria, especially those collected or isolated from soil, contain an important diversity of resistance genes, some similar to genes detected in pathogenic bacteria [14]. In this context, the concept of ‘resistome’ has emerged, which comprises the set of all genes that contribute directly or indirectly to antibiotic resistance in bacteria from the environment, as well as in pathogens of clinical importance [15].

In a strict manner, the resistome consists of: (a) resistance genes from environmental microorganisms, many of which come from the soil and both antibiotic producers and non-antibiotic producers (environmental resistome), (b) resistance genes from pathogenic bacteria (clinical resistome), (c) intrinsic genes present in the bacterial chromosomes that contribute to the resistance (intrinsic resistome), and (d) genes that encode for proteins with metabolic activity that can be precursors to antibiotic resistance genes through evolutionary processes, which have been called proto-resistance genes [16]. Therefore, the resistome is a complex, adaptable, and extensive matrix of genes that act directly or indirectly in blocking the activity of antibiotics [17]. Implications for the emergence of resistance in pathogenic bacteria are significant due to the potential capacity of genes to mobilize through the bacterial pangenome [18].

The resistome is divided in ‘intrinsic resistome or innate’ and ‘extrinsic resistome or acquired’. As Galán et al. mention, in the European Committee on Antimicrobial Susceptibility Testing (EUCAST) expert rules on antimicrobial susceptibility testing, the ‘intrinsic resistome’ is defined as the set of chromosomic genes that participate in the innate resistance, its presence in strains of a bacterial species is independent to the previous exposure to antibiotics, and it is not related to HGT [19,20]. Moreover, the ‘extrinsic resistome’ is defined as the set of genes acquired with simple or multiple changes in the genome, which can be inherited in a stable manner from generation to generation or via HGT [14].

All these genes from the extrinsic resistome can be mobilized by HGT mechanisms to pathogenic and non-pathogenic bacteria from different environments, including humans and animals, among others. The collection of these resistance genes that exist in nature is known as resistome [16].

Based on the proposal of Lekunberri et al., the mobilome refers to mobile elements such as plasmids, integrons, transposons, and insertion sequences (IS) in charge of mobilizing the resistance genes in different environments [21].

The physical movement of DNA is based on a series of ‘cut and paste’ molecular mechanisms capable of manipulating and translocating DNA fragments [22]. The enzymes that participate in these phenomena include recombinases, which facilitate the homolog recombination as part of the encoded machinery of the host that ensures the integrity of the genome; transposases that catalyze the movement and insertion of transposons, which allow the insertion of elements such as resistance cassettes in integrons via site-specific recombination; and resolvases that are DNA endonucleases capable of ‘resolving’ plasmid–plasmid or plasmid–chromosome dimers into monomers in bacteria [23]. Many of these enzymes are encoded by a variety of mobile elements, such as IS, transposons, and integrons, which are capable of facilitating the elimination (also termed excision) or the capturing of genes and accumulation of higher order genetic elements, such as conjugative R plasmids.

In this context, knowledge about these genetic material transfer mechanisms in natural environments gains importance due to implications for several areas, such as the food industry, clinic and hospital environments, and the aquaculture industry, among others. The three main recognized mechanisms of genetic material transfer in natural environments will be described next, considering HGT evidence in bacteria from the genus *Aeromonas*.

## 2. Horizontal Transfer Mechanisms

**Figure 1:**
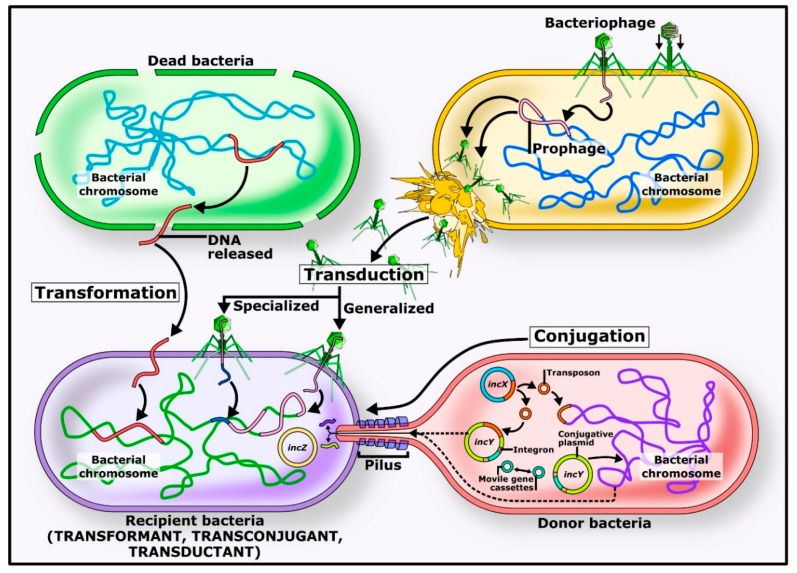
Involved mechanisms in horizontal gene transfer. Transduction, conjugation, and transformation are the main mechanisms by which bacterial species can mobilize and share genetic material with both related and non-related species. These mechanisms imply a pathway for the evolution of bacteria in different environments, allowing them to survive in their niches. A clear example of this is the acquisition of antibiotic resistance mechanisms, virulent traits, and other resources used by the microorganism to guarantee its survival.

Transfer of DNA between bacteria contributes greatly to the evolution and adaptation, due to genes endowing their hosts with resistance to antibiotics and/or metals, pathogenicity, symbiosis, and metabolism of new substrates. The latter can beneficiate the fitness of a bacterium, not only by the utilization of new substrates, but also by allowing the bacteria to thrive in otherwise toxic environments [24]. There are three main mechanisms of DNA transfer described in prokaryotic organisms: transformation, transduction, and conjugation (Figure 1) [25].

Recently, other mechanisms have been described, but these are less described and explored, such as the presence of outer membrane vesicles [26,27], nanotubes [28], and virus-like gene transfer agents (GTAs) [29]. It is important to mention that there are barriers that limit the horizontal genetic transfer, such as the restriction modification system, in which the host bacteria detects the presence of foreign DNA to inactivate it, destroying it with the action of restriction endonucleases, which cleaves dsDNA into fragments that are further degraded by other enzymes. Another barrier is the CRISPR (clustered, regularly interspaced, short palindromic repeats) system, which is comprised of DNA sequences within the genomes of prokaryotic organisms that play the role of a prokaryotic immune system inhibiting the establishment of plasmids and phage infections by the action of the Cas (CRISPR-associated) proteins. In addition to these barriers, there is also surface exclusion, a phenomenon which seems to create an effective barrier against conjugative transfer into bacterial cells that already carry the genes for a closely related transfer apparatus and plasmid incompatibility, a term that will be addressed in the conjugation section of this review [30,31].

### 2.1. Transformation

Bacterial transformation is the genetic alteration in a cell as a result of the direct absorption, incorporation, and expression of exogenous DNA between closely related bacteria, and it is mediated chromosomally by encoded proteins [32]. This foreign genetic material is ‘naked’ and can be present in the environment in which the bacterium thrives, and it can penetrate the bacterial cell membrane when the bacterium is in a ‘competent’ state, either due to lack of nutrients or elevated cell density. In order for the transformation to happen, the DNA must be transferred from the surface to the cytoplasmic membrane and then cross the cytoplasmic membrane through a highly conserved membrane channel [24].

Among aeromonads, an evolutionarily recent high frequency of horizontal gene transfer has been reported [33]. Although natural transformation in the *Aeromonas* genus is not widely known or studied, in 2013, Huddleston et al. performed an analysis of *Aeromonas* species isolated from streams and lakes to determine if the bacterial isolates were generally competent for natural transformation and to characterize the optimal conditions for transformation in a laboratory scheme in order to describe the evolutionary patterns of transformability within the genus. Their findings demonstrated that different strains of *Aeromonas* are naturally transformable under the conditions tested and the optimal conditions for transformation can be correlated to those found in their natural environment. Nevertheless, reported transformation rates were too low (1.95 × 10^−3^ transformants/0.5 µg of DNA) to be considered a molecular tool for this genus, for example, as genome manipulation for library construction or mutation events by transformation [34]. These results suggest that this characteristic of being ‘poorly transformable’ is possibly an intrinsic characteristic of this genus. For the above-mentioned process, optimized transformation protocols have been developed by using electroporation. These works have demonstrated transformation efficiencies in *A. salmonicida* and *A. hydrophila* ranging from 1 × 10^5^ to 4 × 10^2^ transformants/μg of plasmidic DNA [35,36].

These efficiencies are considered low compared to those of Gram-negative bacteria, such as *Pseudomonas aeruginosa* and *Escherichia coli*, in which reported transformation rates were up to 10^7^–10^11^ transformants/μg of plasmidic DNA [37,38]. In addition to this, most aeromonads prefer to accept DNA from close relatives; this is achieved through discrimination that could occur through several mechanisms, such as the donor DNA containing signal sequences recognized by uptake proteins, through the action of restriction enzymes or due to insufficient homology between the donor and the recipient DNA molecules [34]. So far, transformation has not been identified as the responsible mechanism for the acquisition of antibiotic resistance in the *Aeromonas* genus.

### 2.2. Transduction

Another HGT mechanism is transduction, in which DNA transfer is mediated by independently replicating bacteriophages, bacterial viruses that can package segments of host DNA in their capsid, and inject it into a new host when an environmental stimulus triggers cell lysis. When this happens, the new injected genetic material in the cell infected by the virus can be recombined with the chromosomal DNA, generating either a lytic or lysogenic cycle [39]. Bacteriophages can facilitate transfer of other mobile genetic elements within their genomes, such as pathogenicity or genomic islands, and transposons from other bacterial species, due to their genetic topology, with the action of specialized enzymes. These sort of events make recombination a normal occurrence which can contribute to the mosaic structure of phages and the versatility of their genome content [40]. Several reports show the wide variety of *Aeromonas*-specific phages that have been isolated from several sources, mostly environmental ones; two examples of recently identified phages are AhSzq-1 and AhSzw-1, which infect *A. hydrophila* KT998822, both isolated from sea water [41]. Phylogenetical analyses and comparative proteomics show that these phages are two different species and can be grouped as members of the T5 virus genus. These studies also demonstrate the absence of bacterial genetic material of the host in the phage, suggesting that generalized transduction is not a common event in this genus. Other bacteriophages that have been identified and that can be able to inhibit the growth of *Aeromonas* spp. in laboratory conditions during catfish infestation have been reported [42]. These findings show the potential use of these particles as a treatment method to control septicemia caused by *Aeromonas* spp. in the aquaculture field. Phages capable of infecting *A. salmonicida* subsp. *salmonicida* (SW69-9, L9-6, and Riv-10) were isolated from river water and were demonstrated to belong to the *Myoviridae* family by using molecular biology methods and scanning electron microscopy [43]. Although a wide variety of bacteriophages capable of infecting bacteria from the *Aeromonas* genus exists, and there is evidence that shows that transduction is carried out successfully in water environments [44], until now, there is no evidence of a potential role of these phages as elements participating in genetic material transfer events in the bacterial genus.

### 2.3. Conjugation

Lastly, but not least important, there is conjugation, which is considered the main recognized mechanism responsible for genetic material transfer in bacteria and for the emergence of multi-drug resistance in hospital environments and aquaculture, amongst others [45,46]. Conjugation is one of the most active ways of gene transfer, and it is responsible for the propagation of different antibiotic resistance genes in the *Enterobacteriaceae* family, the conjugative plasmids being the most studied mobile genetic elements.

A plasmid is a collection of functional genetic modules organized into a stable entity or ‘replicon’, whose replication must be controlled in consideration of its number of copies, and it may ensure its inheritance by partitioning. Furthermore, the function normally displayed by the plasmid, such as those of replication, maintenance, and conjugative transfer, are mainly dependent on the host factors; therefore, the host’s phenotype may change just by carrying the plasmid [24]. Its genetic organization includes genes that encode for replicative functions and the accessory genes that it harbors. A well-known phenomenon termed incompatibility (*Inc*) must be mentioned, in which some plasmids may not be able to coexist in a cell with other plasmids with the same replication mechanisms [47,48]. In contrast to transformation and transduction, conjugation requires direct cell–cell contact, with the interaction of a highly specialized structure called pilus, which is formed by a protein encoded in the plasmid termed pro-pilin that is part of the tubular structure of the pilus. This cell–cell interaction results in the unidirectional transfer of genetic material (from the donor cell to the recipient cell). This transfer is carried out with the formation of a bridge or conjugative pore that allows the communication between cellular cytoplasm of both cells. Once the recipient cell has acquired the new genetic material, it also acquires the genetic and phenotypic characteristics (encoded in the plasmid) including the transfer characteristics (*tra* genes) [49]. If the plasmid carries structures, such as transposons, integrons, or insertion sequences that have antimicrobial resistance encoded in them, the situation turns more complex due to the acquired multi-drug resistance in these substructures.

Previous research literature shows that the acquisition of genetic material by conjugation in bacteria of the genus *Aeromonas* is carried out successfully. Plasmids of the *IncU* incompatibility group (most frequently identified in *Aeromonas*) were transferred in laboratory-controlled conditions and in in vivo conditions [50,51]. These studies demonstrate that the pRAS1 [*IncU*, class 1 integron (*intI1-dfrA16-qacEΔ1/sul1*), Tn1721 (*TetA*)] and pAr-32 [*IncU*, class 1 integron (*intI1-aadA2-qacEΔ1/sul1*) In6 (*catA2*)] plasmids (both from the *IncU* group) were transferred to clinical *A. hydrophila* strains with 10^−1^–10^−6^, and 10^−3^–10^−6^ frequencies in in vitro and in vivo conditions, respectively. Evidently, the magnitude of plasmid transfer by conjugation in in vivo conditions is affected by several factors, such as native microbiota, the presence of large amounts of organic matter, pH, or relative in situ concentrations of donors and recipients [52]. The findings above demonstrate that biotic and abiotic factors play an important role in genetic material transfer; nevertheless, temperature is one of the most important factors that has not been fully explored. In a previous work by Hernández-Montañez et al., they demonstrated that the transfer of the pRAS1 plasmid was performed in mesophilic strains of *A. veronni, A. media, A. hydrophila*, and *A. caviae*, isolated from frozen fish, with frequencies of 10^−7^–10^−8^ at 8 °C. This evidenced that, even if low temperature is recognized as a factor for the inhibition in bacterial growth for mesophilic bacteria, these can receive extrachromosomal genetic material from psychrophilic bacteria (*A. salmonicida*) [53].

Another example of plasmid transfer in phylogenetically distant organisms is reported by Matsushita et al. in 2018. In their work, they demonstrated the interactive transfer of the plasmid encoding *bla*_IMP-1_ between fecal *E. coli* and environmental *A. caviae* in the ciliate *Tetrahymena* sp. via vesicle accumulation, indicating a pathway for plasmid transfer among bacteria that may be a mechanism for circulation of multi-drug resistant bacteria. In addition to this, there are bacterial species resistant to the phagocytosis of free-living amoebae; when the amoeba engulfs the bacteria, it can thrive inside the host, which encysted protects the internalized bacteria from detrimental conditions found in the environment, playing a role in the selection and transfer of virulence traits among the endosymbionts of the amoeba, facilitating HGT [54,55].

The conjugal transfer of plasmids present in the genus *Aeromonas* to other phylogenetically distant bacteria was reported by Sørum et al. [56]. In their work, bacteria such as *Vibrio cholerae*, *V. parahaemolyticus*, and *Yersinia ruckeri* are described as recipients of plasmids native to *A. salmonicida.* This evidence shows that the flow of genetic material that confers antimicrobial resistance from aquatic bacteria to bacteria of clinical interest can have an important impact on public health.

Antibiotic use in aquaculture generates the appearance of antibiotic resistant bacteria in the environment in which it is performed. This is confirmed by epidemiological and molecular evidence that shows that resistance genes can be transmitted from aquatic bacteria to bacteria capable of producing infections in humans and other terrestrial animals. This shows that aquatic and terrestrial compartments lack restriction barriers in the flux of resistance genes [56,57].

It has been demonstrated that selective pressure in aquatic environments in intensive fish farms lead to the acquisition of antibiotic resistance. Scarano et al. observed an elevated resistance to antibiotics used mainly in aquaculture and human therapy, and the isolated *Aeromonas* spp. strains presented multiple resistance [58].

Due to the wide variety of genetic elements associated to antibiotic resistance in *Aeromonas* strains that can be transferred via conjugation, Piotrowska and Popowska [59] recently performed an insight in the mobilome of *Aeromonas* strains, in which they stated that the mechanisms of pathogenicity in *Aeromonas* spp. are not well understood. This is in addition to recent reports of antibiotic resistant clinical strains that pose a concern about the genus, placing special emphasis on plasmids belonging to different incompatibility groups, most of which carry a number of different transposons, the three classes of integrons, IS elements, or encoded determinants for antibiotic resistance and virulence factors. They defined the mobilome as the whole of genetic elements such as integrons, transposons, conjugative or integrative elements, plasmids, and phages that participate in horizontal transfer events.

For our means, we will focus on those that are related to antibiotic resistance, considering the most commonly administrated antibiotics in the treatment of *Aeromonas* infection, which are levofloxacin, sulfamethoxazole/trimethoprim, amikacin, gentamicin, and ciprofloxacin [60]. As previously mentioned, the genus *Aeromonas* is almost universally resistant to the narrow spectrum of the penicillin group of antibiotics, such as penicillin, ampicillin, carbenicillin, and ticarcillin and susceptible to piperacillin, azlocillin, second- and third-generation cephalosporins, and carbapenems, with most species being susceptible to aminoglycosides, tetracycline, chloramphenicol, trimethoprim sulfamethoxazole, quinolones, and monobactams [61]. Nevertheless, there are exceptions to this depending on the surveyed strains and the type of antibiotic. Such is the case for quinolones, an example of which is in the work of Sen and Rodgers [62], in which they reported antibiotic susceptibility tests performed on over 160 strains of *Aeromonas* spp., their results showed resistance to nalidixic acid, ciprofloxacin, and norfloxacin in an important number of strains, being the three antibiotics evaluated from the quinolones group of antibiotics.

In addition to these data and reports, an analysis of *Aeromonas* genomes searching for acquired resistance genes using the ResFinder database revealed that *Aeromonas* spp. possessed 19 types of resistance genes. In this report, *A. hydrophila* isolates possessed 12 different types of resistance-related genes, being the most commonly predicted resistance mechanism in this study, and *ampH*, *bla*_CEPH-A3_, and *imiH* mediated β-lactamase production [63]. This variability in resistance-related genes and resistance mechanisms has been previously described in well-detailed reviews, with the major mechanism of resistance reported being chromosomal β-lactamases. The work by Janda and Abbott [64] was a review published in 2010 on antimicrobial susceptibility profiles in the genus *Aeromonas* to several classes of antimicrobials. Their findings showed resistance to sulfamethoxazole, cephalosporins, penicillins, and macrolides, some of which are related to the *bla* and *tet* genes, encoded in mobile genetic elements [65,66] or integrons, respectively. These genes are responsible for the resistance to tetracyclines, aminoglycosides, chloramphenicol, and trimethoprim [67,68].

In this matter, production of Ambler class B, C, and D β-lactamases was reported in the genus. Some of these enzymes are metallo-β-lactamases (MBL), such as the CphA type MBL; AmpC β-lactamases, which provide resistance to cephamycins, extended spectrum cephalosporins, and can inactivate β-lactamase inhibitor compounds; and penicillinases. These three are the main β-lactamases harbored by *Aeromonas*, and each strain can produce a maximum of three β-lactamases, which work in a coordinated manner, although these are not the only β-lactamases reported in the genus *Aeromonas*. Extended spectrum β-lactamases (ESBL) producing *Aeromonas* detection is being increasingly reported and, in some cases, infecting *Aeromonas* strains produce a class A β-lactamase of the TEM family of ESBLs, and two more MBLs (VIM and IMP) are found in *A. hydrophila* and *A. caviae* strains. Furthermore, the NDM-1 (*bla*_NDM-1_) gene which encodes for a carbapenemase was detected in the genus, which is of great concern, not only because this enzyme confers resistance to carbapenems and other β-lactam antibiotics, but also because such pathogens are typically resistant to multiple antibiotic classes [61,69].

Antunes et al. [70] detected plasmid-mediated quinolone resistance (PMQR) genes in 21% of the water, sediment, and food samples taken at a trout farm, and in 12% of commercialized trout in three supermarkets. These PMQR (plasmid-mediated quinolone resistance) genes (*qnrS1-S2-S3*, *qnrB7-B19*, *qnrD1*, and *oqxAB*) were detected in *A. hydrophila*, and these are frequently identified in fish farming studies [71,72,73]. Additionally, antimicrobial resistance in *Aeromonas* spp. strains was previously reported by Lamy et al. in 2009 and Aravena-Roman et al. in 2012, whose results indicate that more than half of the tested strains were resistant to the antibiotics groups of antifolates, cephalosporins, and penicillins, as stated by Piotrowska and Popowska [59,74,75]. Lastly, a recent study by Shen et al. in 2018 reported the prevalence of *mcr-3*-positive *Aeromonas* strains from samples of different sources. In their results, they found isolates that harbored colistin resistance mediated by the mobile *mcr-3* gene, showing that the genus may act as a reservoir of this gene, considered epidemiologically important [76].

## 3. Conclusions

The study of the resistome in different levels, such as phenotype, genotype, genomic, and epidemiological level, has turned into an important approach to understand the origin of the antibiotic resistance and its relationship with horizontal gene transfer in the genus *Aeromonas* spp., which is a pathogen related to public health problems. Moreover, with the evidence demonstrating that *Aeromonas* can actively participate in processes of transfer of genetic material (via conjugation) with phylogenetically distant bacteria, the implications for public health become very important. Therefore, it is important to consider the ideal antimicrobial treatment selection for individuals with infections related to multi-drug resistant pathogens.
